# Looking for Myself: Current Multisensory Input Alters Self-Face Recognition

**DOI:** 10.1371/journal.pone.0004040

**Published:** 2008-12-24

**Authors:** Manos Tsakiris

**Affiliations:** 1 Department of Psychology, Royal Holloway, University of London, Egham, Surrey, United Kingdom; 2 Institute of Cognitive Neuroscience, University College London, London, United Kingdom; Macquarie University, Australia

## Abstract

How do I know the person I see in the mirror is really me? Is it because I know the person simply looks like me, or is it because the mirror reflection moves when I move, and I see it being touched when I feel touch myself? Studies of face-recognition suggest that visual recognition of stored visual features inform self-face recognition. In contrast, body-recognition studies conclude that multisensory integration is the main cue to selfhood. The present study investigates for the first time the specific contribution of current multisensory input for self-face recognition. Participants were stroked on their face while they were looking at a morphed face being touched in synchrony or asynchrony. Before and after the visuo-tactile stimulation participants performed a self-recognition task. The results show that multisensory signals have a significant effect on self-face recognition. Synchronous tactile stimulation while watching another person's face being similarly touched produced a bias in recognizing one's own face, in the direction of the other person included in the representation of one's own face. Multisensory integration can update cognitive representations of one's body, such as the sense of ownership. The present study extends this converging evidence by showing that the correlation of synchronous multisensory signals also updates the representation of one's face. The face is a key feature of our identity, but at the same time is a source of rich multisensory experiences used to maintain or update self-representations.

## Introduction

The question of self and identity lies at the heart of human psychology. Experimental research on the physical aspects of self [1] has focused on two domains: self-face recognition, and body-ownership. Even though our physical sense of self is jointly constituted by our physical appearance, of which the face is perhaps its most distinctive feature, and by our sensory-motor body, there has been no direct research link between these two main aspects of selfhood, face and body. Research on self-face recognition has focused on the retrieval of visual representations of one's face [2], while research on body-recognition has investigated how current sensory inflow interacts with motor signals and body-representations [3]. Both research traditions have advanced our understanding of self-face and self-body representations respectively, even though, to date, the interaction between the two has not been investigated. How do I know the person I see in the mirror is really me? Is it because I know the person looks like me, as accounts of visual face perception might suggest, or is it because the mirror reflection moves when I move, and I see it being touched when I feel touch myself, as accounts of body-recognition imply? Or is it a combination of both, and how is this combination determined? Face-recognition studies suggest that visual recognition of stored visual features [4]–[6] and configurations [7] inform self-face recognition. In contrast, body-recognition studies conclude that multisensory integration is the main cue to selfhood [8]–[12]. However, the evidence used in one domain (i.e. body-recognition) may have an unrecognized importance in the other (i.e. self-face recognition). Thus, multisensory evidence for selfhood is widely recognized for bodies, but it may also be important for self-face recognition.

Cognitive psychology has provided detailed accounts of the main principles of face processing, especially when perceiving other people's face, such as hierarchical processing [13], holisitic vs. part processing [14], and processing of identity vs. changeable aspects of faces [15]. However, research on self-face recognition has focused mostly on its neural substrates, especially on the right hemisphere [16]–[20], rather than on the underpinning cognitive processes. The few studies looking at the cognitive processing in self-face recognition emphasize either the presence of view-invariant representations of one's face [7], or the role of mnemonic representations of one's face (e.g. mirror-image) that argue against the existence of robust self-face representations [4]–[6]. Interestingly, the only study looking at self-recognition errors in everyday life [21], reports that approximately half of the normal participants tested had at least once the experience of judging their own face in a mirror or photograph as being the face of someone else. Previous studies on self-face recognition have been largely based on the influence of visual recognition of stored visual features and visual configurations that derive from the perception of other people's faces. These features and configurations are usually only available for our own face when using mirrors. When we look in a mirror we are usually moving or touching the face, and therefore there are multiple proprioceptive, tactile, motor as well as visual sensory cues which are likely to be strong cues to self-recognition. This hypothesis derives from a large body of evidence showing how multisensory signals update cognitive representations of one's body, such as the sense of ownership of body-parts [9], [22]–[23] or whole body [12], the physical appearance of one's body [24] and the sense of agency [11], [25]. For example watching a rubber hand being touched synchronously as one's own unseen hand generates the feeling that the rubber hand is part of one's body [9], [22]–[23]. Asynchronous visuo-tactile stimulation between the two hands does not elicit the Rubber Hand Illusion (RHI). This paradigm suggests that multisensory evidence can be used to produce a sense of self.

**Figure 1:**
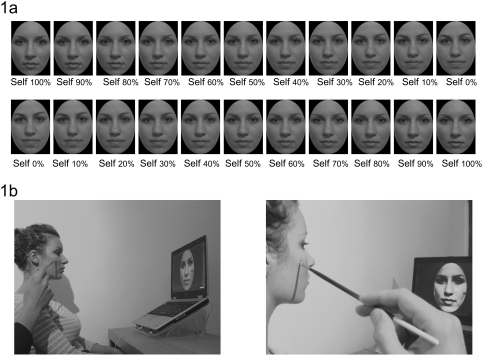
Figure 1 shows the morphing procedure and the direction of morphing (from “self to other” or from “other to self”) displayed in the two types of movies (Fig 1a), and the experimental set-up during the visuo-tactile stimulation (Fig 1b).

**Figure 2:**
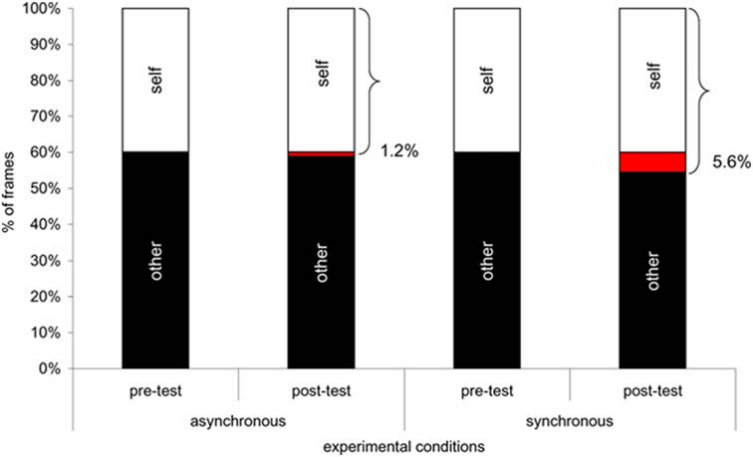
Figure 2 shows the mean % of frames for which the face was perceived to look more like “self” (white bars) or more like “other” (black bars). The areas coloured in red represent the percentage of additional frames that were attributed to the “self” as a result of the synchronous or asynchronous visuo-tactile stimulation.

The present study formally investigates, for the first time, the specific contribution of multisensory stimulation for self-face recognition by using synchronous or asynchronous visuo-tactile stimulation on the face to assess the extent to which *current* multisensory inflow may interact with and alter self-face recognition. Participants watched a morphed face being touched on the cheek with a paintbrush, as if they were looking in a mirror, for 120 sec. The morphed face contained a blending of the participant's facial features (50%) with the features of someone else's face (50%). While participants were looking at the morphed face being touched, the experimenter touched their face with an identical paintbrush in synchrony or asynchrony on the same location (see Methods, and Figure 1). Before and after the exposure to this multisensorial combination of felt touch and vision of touch, participants performed a self-face recognition task [17]. Participants watched movies that contained the whole morphing sequence in 1% morphing transitions, either from other to self (i.e. from 0% self to 100% self), or from self to other (i.e. from 100% self to 0% self, see Figure 1a). They were instructed to stop the movie when they felt that the face was starting to look more like self than other, or vice versa, depending on the morphing direction displayed in the movie. The points at which the participants stopped the movies were used to calculate the percentage of frames that were judged as belonging more to the participants' own face across conditions (see Figure 2). The analysis focused on the differences in the chosen points at which participants stopped the movie before and after their exposure to multisensory stimulation.

## Results

The mean values (see Table 1) were submitted to a 2×2×2 ANOVA, with the factors of direction of morphing (i.e. from self to other, or from other to self), the mode of visuo-tactile stimulation (synchronous or asynchronous), and the judgment (pre- or post-test). The main effect of direction of morphing (F(1,11) = 125.44 p<0.05), of stimulation (F(1,11) = 10.46 p<0.05) and judgment (F(1,11) = 13.71 p<0.05) were significant. The two-way interaction between judgment and direction was not significant (F(1,11) = 1.8, p>0.05). The two-way interaction between direction and stimulation was not significant (F(1,11) = .33, p>0.05). Importantly, the two-way interaction between judgment and stimulation (F(1,11) = 17.93, p<0.05) was significant, while the three-way interaction (i.e. direction×judgment×stimulation, F(1,11) = 1.88 n.s.) was not significant.

**Table 1:** The mean % of frames where the face was perceived to look more like “self” than “other” across conditions.

	“Other to self”		“Self to other”		Grand Mean	
	Pre-test	Post-test	Pre-test	Post-Test	Pre-Test	Post-test
Asynchronous Stimulation	36.8% (*1.1*)	38.2% (*1.1*)	43.2% (*0.5*)	44.1% (*0.5*)	40.0% (*0.7*)	41.2% (*0.6*)
Synchronous Stimulation	36.4% (*1.2*)	44.3% (*1.7*)	43.9% (*0.9*)	47.0% (*1.2*)	40.1% (*0.5*)	45.7% (*1.1*)

The main effect of morphing direction was highly significant as noted above. In the “self to other” direction, participants stopped the movie after 44 frames on average, while in the “other to self” direction, they stopped the movie after 62 frames on average. Thus, participants stopped the movie earlier when they had to judge if the face looked more like other , and they stopped the movie later when they had to judge if the face looked more like self. Even though, the two morphing directions yielded statistical differences, the observed pattern is consistent with less than 50% of frames being “classified” as belonging to self across both conditions (44 frames for “self to other”, and 38 frames in the “other to self”). The pre-tests in the main experiment show a trend to judge the seen face as looking more like the other than self. This bias may reflect a bias in self-recognition that has been previously reported in self-recognition studies [17]–[18], but it may also reflect a more general familiarity bias. Both the main experiment and the control experiment (see below) suggest that participants are particularly sensitive to changes in familiar faces. However, the important finding of the present study is that synchronous stimulation significantly reduces this bias, even if the average frame in post-tests after synchronous stimulation contains objectively more self than other. Because it is only the 2-way interaction between judgment and stimulation that is significant, it seems unlikely that the main effect of morphing direction can account for the differences between pre- and post judgment for each level of visuo-tactile stimulation.

Planned comparisons between pre and post-test judgments showed that synchronous but not asynchronous stimulation resulted in a significant change in the self-recognition judgments when participants saw the “self to other” morphing (t(11) = 2.6, p<0.05, 2-tailed), the “other to self” morphing (t(11) = 2.95, p<0.05, 2-tailed), and also when we compared the mean values collapsed across the two morphing directions (t(11) = 4.27, p<0.05 2-tailed). Differences between the pre-test and post-tests with asynchronous stimulation were not significant for any morphing direction (t(11) = 1.1, p>0.05 , t(11) = 1.2, p>0.05 and t(11) = 1.7, p>0.05 respectively).

In a follow-up analysis, the shifts in self-face recognition as a result of multisensory stimulation were estimated as the difference between judgments in the post-test and the judgments in the pre-test across the synchronous and asynchronous stimulation conditions. Planned comparisons revealed significant differences between synchronous and asynchronous shifts for the “self to other” direction of morphing (t(11) = 2.3, p<0.05, 2-tailed), the “other to self” direction of morphing (t(11) = 2.5, p<0.05, 2-tailed), and also when we compared the mean shifts across conditions (t(11) = 4.2, p<0.05, 2-tailed). This analysis confirms the hypothesis that it is not the mere presence of multisensory stimulation that alters self-face recognition, but instead it is only the synchronous visuo-tactile stimulation that changes self-face recognition over and above the mere presence of visual and tactile stimulation.

To ensure that this effect was self-specific and not simply due to the presence of synchronous visuo-tactile stimulation, 6 new participants performed a control experiment in which their own face was replaced by the face of a highly familiar face across all phases of the experiment. Participants completed two synchronous and two asynchronous blocks with the same methods as in the main experiment, apart from the fact that the participants' own face was replaced by the face of a highly familiar face across all phases of the experiments, while the other face was that of an unfamiliar person. The points at which the participants stopped the movie were used to calculate the percentage of frames that were judged as belonging more to the familiar face across conditions (46.5%±2.3 S.E.M. for pre-test/asynchronous, 47.1%±1.8 S.E.M. for post-test/asynchronous, 47.4%±2.3 S.E.M. for pre-test/synchronous and 47.4%±2.1 S.E.M. for post-test/synchronous). Neither main effects nor the interaction were significant (F(1,5) = .44, p = .53 for the type of stimulation, F(1,5) = .38, p = .56 for the judgment, and F(1,5) = .13, p = .72 for the interaction). The results revealed no significant changes in the recognition task as an effect of synchronous stimulation, suggesting that the observed effect in the main experiment cannot be accounted simply by face-familiarity.

The participant's subjective experience was not systematically assessed with questionnaires after each block to avoid suggestibility. During informal debriefing at the end of the experiment, some participants reported that during synchronous stimulation they felt as if they were looking at a mirror but not seeing exactly their own face (n = 5), or that they were looking themselves at the mirror (n = 2), or a video (n = 2), or that the experience was uncanny in the sense that the touch they felt matched exactly the touch they saw on a face that “was, and at the same time wasn't” their own face (n = 2). When asked about the difference between synchronous and asynchronous stimulation, participants reported that the experience of touch on their own face was more salient in the synchronous conditions (n = 5), and that the synchronicity of the two events established a “strong link” between the face on the screen and their own face (n = 4). For the asynchronous condition, participants felt that the touch they saw on the screen would predict the touch on their face (n = 7), or that the two events were not related (n = 4). One participant opted out of debriefing.

Overall, the behavioral results of the main experiment show that after synchronous stimulation, participants accepted as self-stimuli, faces that were significantly more morphed towards the other person than those accepted after asynchronous stimulation. Faces containing an average 5.6% more of someone else's face were judged as ‘self’ after synchronous stimulation compared to before (see Figure 2). This pattern reflects a statistically significant shift in the internal representation of one's own face, due to synchronous visual-tactile stimulation.

## Discussion

This experiment investigated for the first time how current multisensory inflow may interact with, and possibly alter self-face representations. The results suggest that a strong correlation between synchronous visual and tactile signals influences self-face recognition over and above the mere presence of multisensory stimulation, and it may alter internal representations of one's own face, analogous to the effects of multisensory stimulation for body-ownership. Synchronous multisensory stimulation can update cognitive representations of one's body, such as the sense of ownership of body-parts [9], [25] or whole body [12], and the physical appearance of one's body [24]. The present study extends this converging evidence by showing that multisensory signals also update the representation of one's face.

A recent review highlights both similarities and differences in the way we process faces and bodies [26]. While, detection of faces and bodies is underpinned by distinct cortical areas, presumably because it depends on the recruitment of basic visual categorization processes, recognition of faces and bodies requires more complex processes such as configural analysis and identification. Face- and body-perception are consistently linked to activity in the fusiform face area [27] and the extrastriate body area [28] respectively, while recognition of one's face or body is often linked to multisensory areas, predominantly on the right hemisphere. Accumulating neuroimaging data report activations in the insular and parietal cortices associated with body-ownership [23], [29], agency [30]–[31] and self-face recognition [32]–[33], suggesting that all these aspects of the self may share a partially common neural substrate in the right hemisphere. Interestingly, the few case studies of delusional misidentification (for a review see [34]) following focal brain lesions report lesions in the right hemisphere. In terms of the underlying cognitive deficits of delusional misidenification, different accounts have been suggested in the literature, such as prosopagnosia, mirror agnosia, disordered facial body schema, impaired facial processing and visuso-perceptual deficits. More recently, hypnotic suggestion was successfully used to induce delusional mirrored-self misidentification in healthy volunteers, suggesting that top-down processes may also underlie self-identification [35].

Interactions between seeing one's own face and multisensory stimulation have also been reported previously. For example, seeing one's own face being touched enhances tactile perception on the face [36]. In addition, being exposed to one's own odour, or seeing/hearing one's own name has been shown to facilitate self-face recognition [37]. However, these previous studies did not investigate the role of crossmodal facilitations or multisensory stimulation for maintaining or updating a representation of one's face. The present study shows that a shared multisensory experience may update the internal representation of one's face, in the same way that multisensory stimulation in the rubber hand illusion may update the internal representation of the physical appearance of one's own hand. A recent study [24] shows that incorporation of the rubber hand into the body image affects the similarity that participants perceived between their own hand and the rubber hand. Specifically, participants' similarity ratings were correlated with the subjective experience that the rubber hand was becoming more like their own hand, but not the converse [24], [see also 38]. In a similar way, the results on self-face recognition also point to the same direction of change because after synchronous multisensory stimulation participants accepted as self-stimuli faces that were more extensively morphed. Such changes in the perceived similarity of body parts reported in that study and of faces as reported in this study may explain the ways in which self-representations are constructed and updated.

Ontogenetically, the existing evidence on self-recognition suggests that an implicit bodily sense of self appears before explicit self-face recognition in the mirror (see “the rouge task” [39]). Newborn infants can discriminate between endogenous and exogenous tactile stimulation, and by the 3rd month, infants can detect visuo-proprioceptive incongruencies [40]. Explicit mirror self-recognition occurs between months 14th–18th [39]. Therefore, an implicit body-awareness that depends on the efficient integration of multisensory signals precedes the explicit recognition of one's face in the mirror that seems to be constructed by the assimilation of congruent multisensory signals. In addition, multisensory signals can also be used to update self-representations by assimilating external events into a pre-existing body image [24], [38]. The changes induced as a result of multisensory experience affect mainly the representation of one's self and body in relation to other people or bodies. It is therefore plausible that even when we feel physically dissimilar to each other, shared multisensory experiences can make us feel to be more similar. As recent developmental models suggest [41]–[42], a basic “like me” process whereby percepts of other people's actions, appearance and identity are interpreted in terms of one's own actions, appearance and identity, may form the basis of intersubjectivity and social cognition. Other situations such as joint action [43], and automatic imitation [44] also provide multisensory inputs that are comparable with the inputs used in the RHI and the present study. Future studies can actively exploit such experimental paradigms to investigate whether changes in self-representations can be followed by specific changes in the way we perceive other people with whom we share multisensory experiences.

The experience of looking into one's face in the mirror is accompanied by a continuous integration of tactile and proprioceptive events perceived on one's face and visual events perceived on the mirror-reflection. These sensory inputs are assimilated in a pre-existing visual representation of one's face. In line with previous studies, the present findings suggest that visual capture of touch can update representations of one's physical appearance. The reported effect provides direct evidence that our body image, including the representation of one's face, is not solely derived from stable representations, but instead these representations are susceptible to current multisensory evidence. The face is a key feature of our identity, but at the same time is a source of rich multisensory experience. Shared multisensory experiences may be used to maintain or update self-representations and also change the way we perceive other people.

## Materials and Methods

### Pre-processing

A digital photograph of the participant was taken in a session prior to the experiment. The participant's face in the photograph was mirror transposed, and a black template was used to remove non-facial attributes (e.g. background, hair, ears) with Adobe Photoshop CS4. A computerized morphing procedure implementing a mesh warping algorithm (Abrasoft Fantamorph, www.fantamorph.com) was used to merge each participant's face with an unfamiliar face (same sex, same age+/−1 year) in 1% steps, resulting in 100 images (from 0% self to 100% self, or from 100% self to 0% self) with graded blending of the facial features of the two faces.

### Experimental Procedure

In the experimental session, participants were first asked to watch a movie consisting of 100 frames (Pinnacle Studio Software). Each frame represented a 1% incremental change from one face to another, from “0% self to 100% self” (i.e. “other to self” direction) or from “100% self to 0% self” (i.e. “self to other” direction). For the movies with “other to self” direction of morphing, participants were asked to press a key, with their right index finger, as soon as they perceived the face to look more like “self” than “other”. For the movies with “self to other” direction of morphing, participants were asked to press the key as soon as they perceived the face to look more like “other” than “self”. The responses were logged and they served as a baseline measure of self-face recognition. Participants received prior training on this task. They watched movies where the image of Tony Blair was morphed into George Bush or the reverse and they stopped the movie at the point where the face was more like Blair than Bush or the reverse.

Following this pre-test, participants were asked to look at the screen placed in front of them and observe the projected movie. The movie showed a paintbrush touching a morphed face on the cheek every 2 sec either in synchrony or asynchrony with respect to the touch delivered on the participant's face. Tactile stimulation on the participant's face occurred every 2 seconds across both synchronous and asynchronous conditions. The asynchrony between visual and tactile stimulation in the asynchronous condition was 1 second. Therefore, the amount of stimulation across synchronous and asynchronous stimulation was the same. Each stroke covered a distance of approximately 2 cm on the face. The morphed face displayed in the movie contained 50% of the participant's face and 50% of the face of an unfamiliar person, matched for gender and age. As soon as the image appeared on the screen, tactile stimulation was delivered on the participant's right cheek through an identical paintbrush. Visuo-tactile stimulation was delivered manually on a specular congruent location on both faces (see Figure 1b). The experimenter listened through earphones the audio file of the pre-recorded movie to pace his tactile stimulation in synchrony or asynchrony with the metronome that was used to deliver the tactile stimulation shown in the movie. Thus, while the participant was looking at a single morphed face, she was being touched on the same facial location either at the same time (i.e, synchronous visuo-tactile simulation) or at different time onsets (i.e. asynchronous visuo-tactile stimulation). Each movie and stimulation period lasted for 120 secs.

At the end of the stimulation period, the morphed image disappeared, and participants watched again a movie consisting of the same 100 frames as in pre-test. Participants were asked to stop the movie as before, depending on the direction of morphing. The point at which participants stopped the movie represents the effect of the prior (synchronous or asynchronous) multisensory stimulation on self-face recognition.

In total, each participant performed 8 blocks (4 with synchronous and 4 with asynchronous visuotactile stimulation). In each block, the movie in the pre- and post-test displayed the same direction of morphing (i.e. from “other to self” or the reverse), and participants performed 4 blocks with each direction of morphing. Each pre- or post-test movie displayed the same 100 frames, but the duration of the movie was varied to prevent participants from giving the same response. Thus, the movies lasted for either 50 sec or 100 sec. The order of blocks was randomized across participants, and a five minutes break was interleaved between blocks.

### Participants

12 participants with normal vision (8 female, mean age 22.6) participated in the main study. 6 additional new participants (all female, mean age 23.8) participated in the control experiment. Participants were informed that the study was designed to investigate how shared multisensory experiences affect our representations of people's faces, and no specific mention to self-face recognition was made. During debriefing, participants were informed that the aim of the study was to investigate how multisensory input can influence self-face recognition. The study was approved by the Departmental Ethics Committees, Department of Psychology, Royal Holloway, University of London.
